# Some Methods of Extirpating Pulps, and Subsequent Treatment

**Published:** 1900-09

**Authors:** James B. Locherty


### SOME METHODS OF EXTIRPATING PULPS AND SUBSEQUENT TREATMENT.[1]

BY DR. JAMES B. LOCHERTY.

Mr. President and Gentlemen,—The many cases indicating the employment of arsenous acid preparations, or some other method for the purpose of destroying the dental pulp, owing to the extreme sensitiveness of it, are in a measure some excuse for the numerous papers which have been presented before the different societies from time to time, and with your kind indulgence I will submit the following paper, in which I shall endeavor to set forth as briefly and concisely as possible some of the methods adopted in extirpating pulps, theories regarding such methods as to the danger, if any, which might possibly attend the use of certain agents employed for that purpose, and, finally, some considerations respecting the subsequent treatment after the removal of the pulp.

For convenience the methods will be classified as medicinal and surgical. Under the former, arsenous acid preparations will be considered, while in the other, or surgical method, cocaine, separately or in combination with some other agent, will be mentioned.

As to the medicinal treatment: For many years arsenous acid preparations have had many advocates, and those who have had a long and varied experience in the use of them have, by making applications judiciously, destroyed the pulp in a practically painless manner, while, again, many practitioners have contended that its employment should be dispensed with and a suitable substitute used.

---

[1] Read before The New York Institute of Stomatology, April 3, 1900.

Several months ago a paper appeared in the *Dental Cosmos* on the uses of arsenous acid, by an advocate, in which he contended that it was "less painful and less dangerous to dental tissues and the general health of the patient than some of the means suggested as substitutes." He also stated that "it is a successful and conservative method, and although it has been abused in some instances through carelessness and ignorance, yet an intelligent application of it is legitimate and scientific practice;" and among other things was a formula for an arsenous acid paste, also the therapeutic actions of the respective drugs employed therein and the satisfactory results obtained; and, in concluding, the writer stated that if, through carelessness of the patient or from other cause, the peridental membrane becomes inflamed, by applying liquid dialyzed iron the toxic effect would cease, as the arsenous acid would form arsenite of iron. While another practitioner, opposed to the use of arsenous acid, stated that the practice of applying poisonous escharotics, such as arsenous acid, should always be avoided, with the appliances and facilities now at the command of every dentist. "The use of such agents is wholly unnecessary, and especially so when it is considered that the most serious results follow their use in some instances, in either the immediate or remote future, and especially in the cases of those highly susceptible to the poisonous influences of arsenous acid or similar agents; specific results in some instances occurring in twenty-four hours. The results, however, may in other cases be delayed for weeks or months before manifesting any action in a marked manner."

Now, the opinion of the gentlemen just quoted are simply illustrations of the widely different views which are maintained on this subject, and while we are loath to believe that such opposite views are universally held by all the members of our profession to-day, still there are a vast number who, according to their past experiences, would readily take sides with one of the above views; but were one to look at the subject dispassionately, the conclusion would probably be, that in both the medicinal and surgical method there is much to be commended, allowing that some particular method be adopted which has stood the test of time and experience successfully, notwithstanding that the physiological and pathological action of any particular agent may be fraught with much danger. But in the adoption of a plan for the extirpation of the dental pulp, the first consideration should be the use of that method which will be the more scientific; that is, not only should the manner of application be thoroughly understood, but also certain considerations regarding either the physiological or pathological action of the agent or agents employed in the treatment; also as to which is the more favorable condition present for the future preservation of the tooth-structure after the extirpation of the pulp.

In considering the medicinal method, the principal agent being arsenous acid, a standard work informs us that it is a powerful caustic; an internal dose is a very severe gastro-intestinal irritant, in minute doses a gastric stimulant causing dilatation of the gastric vessels. As to its effect upon circulation, it says, "The rapidity and force of the pulsation is lessened until it finally stops. It is eliminated by the urine, the alimentary canal, the skin, the saliva, the milk, and even the tears; it may be found many years after death in the bodies of those who have taken it in life. Dose, one-sixtieth to one-tenth of a grain. As a drug it certainly in some way alters the condition of the sufferer, and is vaguely called an alterative." Of course, the above is in general therapeutics. Its action, however, may be considered a purely local one when confined to the pulp-cavity and used in infinitesimal quantities. It is probably owing to its being a strong irritant that many practitioners believe that its action distends the vascular tissues, which causes the pulp to be destroyed by strangulation. One writer in *Items of Interest,* for May, 1898, says that, "most vasomotor nerve-fibres of the sympathetic system having been found to possess constrictor and dilator functions, when injured or acted upon by certain medicinal agents the vasodilator function persists for some time longer than the vasoconstrictor action;" he suggests that, "may we not find in this fact an explanation of arsenous acid in the destruction of the pulp?" "the first action being the paralyzation of the vasoconstrictor fibrils; the vasodilator function continuing, the blood-vessels becoming rapidly engorged, the pulp dies from exhaustion." While another theory is that arsenous acid forms a chemical combination with the hæmoglobin of the blood, called arsen-hæmoglobin, and through its toxic power causes structural degeneration of the cellular elements, followed by the death of the tissues as far as the arsenous acid has penetrated and formed arsen-hæmoglobin (*Dental Cosmos,* September, 1899). Notwithstanding our absence of precise knowledge as regards arsenous acid, it has certainly performed its mission in the destruction of the pulp; but it is owing to its dangerous properties, and also that permanent relief may be given a patient suffering from a congested or inflamed pulp, a relief not always attended, when arsenous acid is applied, until a certain period of time has elapsed,—a sufficient period to cause devitalization of pulp,—that has caused us to abandon the use of arsenous acid preparations and use the surgical method in its stead, believing it to be a more scientific method.

The employment of cocaine, either separately or in combination, is, of course, as you all well know, not absolutely without danger. As to its action, we are informed that "it constricts the arterioles, that a two to ten per cent, solution is sufficient to anæsthetize the sensory nerves, while a larger dose is necessary to similarly affect the motor nerves." "As to its action upon the respiratory centre, it first stimulates it, so that the rapidity and depth of respiration are increased; but soon depression of the centre follows, the respiratory movements become feeble, and death takes place from asphyxia. There are, however, several antidotes suggested in the use of hydrochlorate of cocaine, suprarenal extract (as suggested by Dr. Dawbarn), whiskey, or strychnine, but in the minute doses and the manner in which it may be applied locally when necessary for the extirpation of the dental pulp, the toxic power of cocaine may be reduced to a minimum; in fact, experience has taught us that, in the exercise of extreme caution in its application, not the slightest fear need be entertained as to any serious conditions likely to arise in its use when extirpating dental pulps, if the following precautionary measures be adopted, which are, that the rubber dam be used in every instance where practicable; conditions existing where it is impossible to adjust the rubber dam, other measures must be employed in order to confine the agent to the tooth or cavity in question and avoid its being absorbed by the mucous membrane, the great point being to exclude all moisture or exudations from the surrounding tissues.

Several methods are employed in applying cocaine, such conditions, for instance, as the position of the tooth, location and extent of cavity governing the special method to be adopted. A few typical cases will serve to illustrate.

Case I.—Upper right cuspid; condition of mouth wherein it was advisable to extirpate pulp; applied rubber dam and ligatured it to tooth; ground off enamel on palatal portion about on direct line with pulp. When reaching dentine used drill (spear point) for short distance, then applied, in this particular instance, vapocaine (an etherealized solution of cocaine), although a saturated solution of cocaine, or even ten per cent. solution, would answer the purpose, but we believe it is not readily absorbed. After a few moments the drill was again used until sensitive dentine was reached, when the application was repeated, and with an ordinary amalgam plugger applied pressure to small piece of unvulcanized rubber to the drilled cavity, believing that in so doing the cocaine is made to penetrate the dentine more rapidly. After allowing ample time to elapse for the obtunding of the dentine, used cocaine on approaching sensitive dentine, again applying solution, thus continuing cautiously until enabled to enter pulp-chamber with practically no more pain than a patient would experience in excavating a slightly sensitive tooth. The condition present upon reaching the pulp seems to be that the cocaine has constricted the blood-vessels in it, so that it not infrequently happens that we are able to enter the pulp-cavity without causing any hemorrhage whatever. The hypodermic syringe is then used, and a ten per cent. solution of hydrochlorate of cocaine, or saturated solution, injected, say a drop or so, and allowed to more completely anæsthetize the pulp. After a few more moments had elapsed, was enabled to enlarge, by means of rose bur, an artificial cavity to pulp; then by aid of Donaldson's nerve-broach, without handle, taken between thumb and forefinger, and by a rotary or rather twisting or screwing motion, was enabled to pass it up alongside of pulp and extract it in a painless manner. The above instance is a typical case, the patient being of a highly nervous temperament, the operation occupying about thirty minutes.

Case II.—A Miss N.; upper left second bicuspid, cavity distal approximal, pulp slightly exposed; applied rubber dam and ligatures to necessary teeth and then adopted the following method: Used crystals of hydrochlorate of cocaine to pulp, allowing ample time for absorption, then by means of instrument worked crystals into pulp-chamber and applied a piece of unvulcanized rubber, using pressure to hold it there for a few moments; and was then enabled, after enlarging the pulp-canal, to remove the pulp. For this method we are indebted to Dr. Watkins, of Montclair, who learned of it, we believe, at the National Convention a few years ago. We must confess that the method of procedure did seem rather heroic, but it is astonishing with what rapidity a pulp may be extirpated, and at the same time with but little pain, especially if we allow from twenty to thirty minutes for absorption of crystals of cocaine.

CASE III.—Mr. R.; lower left first molar pulp; owing to exposure, cavity slightly inflamed, in this instance situated in the mesial portion, near cervical margin of tooth. In this instance did not apply rubber dam, but by means of bibulous papers and varnish was enabled to keep surrounding parts quite free from moisture. Upon removing filling on mesial approximal portion, was enabled, by means of saturated solution of hydrochlorate of cocaine, to reach pulp-cavity in a similar manner as referred to in Case I.; then applied sulphuric acid C. P. and cocaine, equal parts, to pulp, applying pressure by the aid of unvulcanized rubber; the sulphuric acid, having a great affinity for water, seems to disintegrate the pulp after causing some little pain, not sufficient, however, to cause the patient any great discomfort. By thoroughly enlarging the pulp-chamber I was able to remove its contents, and then, after washing out in a manner shortly to be described, applied the above solution to the individual canals, applying pressure in the manner as before described, and removed contents from canals.

Case III. is cited as an instance of the great vitality which some nerves seem to possess, for the tooth had not only troubled the patient for some months, but it was also difficult to extirpate—the sulphuric acid C. P. with cocaine is a formula that I would not unhesitatingly recommend, for there is a possibility of some pain attending its application; at least, that has been my experience with it, although it will be found excellent for extirpating so-called nerve-stumps or filaments at the end of root-canals; but in Cases I. and II. the method may be adopted and applied with uniformly excellent results, the duration of time extending from ten minutes to an hour, depending upon existing conditions. Of course, many other methods have been used, such as eucaine, nitrous oxide gas, ethylchloride, cataphoric and air-pressure, with various success, although having used cocaine as described in the above cases, especially I. and II., with results that have been decidedly satisfactory. I am perfectly willing to continue the treatment until a superior method, especially as to time and safety, may be suggested as a substitute. As to the subsequent treatment in instances where the pulp has just been extirpated and the rubber dam is still in position, the pulp-canal is cleansed by forcing warmed distilled water, by the aid of the hypodermic syringe, until the contents are thoroughly removed, then partially wiping the roots. Sulphuric acid, fifty per cent. solution, is applied,—*i.e.*, equal parts alcohol and sulphuric acid,—and a few drops of oil of cassia, care, however, being exercised so as not to force it beyond the apical foramen. After applying this agent for a few moments the canal is dried by hot air, the absolute alcohol and hydronaphthol are applied, and the canal filled with chloropercha and gutta-percha cones. This is the general treatment, although other methods are sometimes adopted.

The improvement in the treatment of root-canals has been as marked as other methods have along similar lines in matters pertaining to our profession, and all appreciate the fact that it is absolutely essential to surgically cleanse the canal. There have been many excellent instruments devised for that purpose, in the shape of improved highly tempered nerve-broaches and nerve-canal drills, to remove organic matter which might cause trouble at a future time, yet the physical characteristics presenting themselves in the form of tortuous or irregular canals confront us with a condition in which it is not always practicable to thoroughly remove the pulp in its entirety. What is the best method of treatment to adopt? Of course, if we can employ an agent which will chemically change the remaining contents of the canal into a permanent sterile condition, that will no longer be a source of much annoyance. We still have to consider one or two points suggesting themselves, which are the albuminous matter in the dentinal tubuli, the methods used in attempting to place these tubuli in an aseptic condition, the manner in which agents act producing asepsis, and their germicidal power and stability.

An authority some time ago stated that investigation tended to show that the absorption of liquids into the dentinal tubuli was by osmotic action, and that coagulants soluble in water would diffuse through tooth-structure, and also that oleaginous non-coagulants also passed through the tooth-structure, although slowly in the presence of water in serum albumin; farther on stating that the function of water in the tubuli was necessary as a carrier of medicinal agents.

This point was simply made touching upon what we believe the importance of moisture or water being present in the tubuli, at least, of the root, to assist us in medicating tooth-structure, except in instances where air pressure is used. As to the antiseptic properties of agents, it is well known that under different conditions their actions vary greatly, as, for instance, the action of iodoform, in culture mediums, being an indifferent substance, while in diseased condition it manifests strong antiseptic qualities. "The power of antiseptics depends upon the temperature at which they act, the medium in which they are dissolved, the strength of the solution, the time given them to act, and micro-organisms present in the substances to which they are added." This being the case, it behooves us to use such antiseptics in our treatment as will be quick in action, at the same time being permanent or stable, and while I believe we have not yet been able to find an antiseptic with the latter quality, yet we are fortunate in having many at our command to-day, the application of which, if the proper surgical work be performed at the beginning, will, in almost every instance, place the canal in a thoroughly aseptic condition; then by a suitable root-filling, the patient will have a tooth which will cause him in a vast majority of instances no further trouble in this direction.

